# How to Address the Adjuvant Effects of Nanoparticles on the Immune System

**DOI:** 10.3390/nano10030425

**Published:** 2020-02-28

**Authors:** Alexia Feray, Natacha Szely, Eléonore Guillet, Marie Hullo, François-Xavier Legrand, Emilie Brun, Marc Pallardy, Armelle Biola-Vidamment

**Affiliations:** 1 Université Paris-Saclay, Inserm, Inflammation, Microbiome and Immunosurveillance, 92290 Châtenay-Malabry, France; alexia.feray@universite-paris-saclay.fr (A.F.); natacha.szely@universite-paris-saclay.fr (N.S.); eleonore.guillet@universite-paris-saclay.fr (E.G.); marie.hullo@gmail.com (M.H.); marc.pallardy@universite-paris-saclay.fr (M.P.); 2 Université Paris-Saclay, CNRS, Institut Galien Paris Sud, 92296 Châtenay-Malabry, France; 3 Université Paris-Saclay, CNRS, Institut de Chimie Physique, 91405 Orsay, France; emilie.brun@universite-paris-saclay.fr

**Keywords:** amorphous silica nanoparticles, monocyte-derived dendritic cells, MoDCs, maturation, danger signal, DC:T-cell co-culture, in vitro models and methods, nanotoxicology

## Abstract

As the nanotechnology market expands and the prevalence of allergic diseases keeps increasing, the knowledge gap on the capacity of nanomaterials to cause or exacerbate allergic outcomes needs more than ever to be filled. Engineered nanoparticles (NP) could have an adjuvant effect on the immune system as previously demonstrated for particulate air pollution. This effect would be the consequence of the recognition of NP as immune danger signals by dendritic cells (DCs). The aim of this work was to set up an in vitro method to functionally assess this effect using amorphous silica NP as a prototype. Most studies in this field are restricted to the evaluation of DCs maturation, generally of murine origin, through a limited phenotypic analysis. As it is essential to also consider the functional consequences of NP-induced DC altered phenotype on T-cells biology, we developed an allogeneic co-culture model of human monocyte-derived DCs (MoDCs) and CD4+ T-cells. We demonstrated that DC: T-cell ratios were a critical parameter to correctly measure the influence of NP danger signals through allogeneic co-culture. Moreover, to better visualize the effect of NP while minimizing the basal proliferation inherent to the model, we recommend testing three different ratios, preferably after five days of co-culture.

## 1. Introduction

With the widespread production and use of nanomaterials, the growing exposure of workers but also of consumers in everyday life raises concerns about potential health risks [1], strengthening the need for relevant methods to evaluate their immunosafety [2]. Assessing how engineered nanoparticles (NP) could interact with the immune system [3,4] and more specifically could promote the development of allergic diseases or exacerbate the severity of established allergic conditions is still a matter of concern. Experimental in vitro models must be developed to address and to monitor these effects [5]. The challenge is to select the most relevant cells and endpoints using a simple, robust and representative model.

Most of the work regarding NP-induced immunomodulation has been focused on the activation of key immune cells [6,7,8]. Innate immune cells such as macrophages and dendritic cells (DCs) are predominantly exposed to nanomaterials due to their phagocytic properties and to their relative abundance at the entry sites such as skin, airways or the gastrointestinal tract [9]. Indeed, DCs patrolling the blood or residing in peripheral tissues actively capture NP [10].

Two main differentiation stages are currently described for DCs, namely “immature” and “mature” [11]. As sentinel cells of the innate immune system, immature DCs encounter and internalize invading pathogens, but are also tissue scavengers, capturing apoptotic or necrotic cells. Their functional maturation is a process, initiated by signals from the microenvironment and characterized by the acquisition of fundamental properties: antigen processing and presentation, migration and ability to activate antigen-specific naïve T-cells in secondary lymphoid organs. Indeed, upon maturation, DCs increase the expression of major histocompatibility complex-II (MHC-II) and co-stimulatory molecules, express higher levels of chemokine receptors and secrete cytokines, working in concert to stimulate specific immune responses. DCs maturation can be triggered by the recognition of pathogen-associated molecular patterns (PAMP) or endogenous “danger” signals called damage-associated molecular patterns (DAMP) through a battery of pattern-recognition receptors (PRRs) expressed both outside and inside of the cells [12]. The “danger theory” was proposed by Polly Matzinger twenty-five years ago [13] to explain how the immune system could inappropriately “see” innocuous antigens such as allergens or food antigens as dangerous, even though they are not. The “danger signals” would act on DCs, in aseptic conditions, in a similar manner to the microbial molecular patterns. Therefore, the question to be addressed is whether engineered NP could act as danger signals, as previously suggested for ultrafine or diesel exhaust particles [14] and could be considered as NAMPs (nanoparticle-associated molecular patterns) [15,16,17].

As terminally differentiated post-mitotic cells, primary DCs isolated from blood are available in limited amounts. Because no stable cell line fully recapitulates the immunological features of primary DCs [18], studies on NP interactions with DCs use either murine bone marrow-derived DCs (BMDCs) [6,19,20] or more rarely human blood monocyte-derived DCs (MoDCs) [21,22,23], which can both be differentiated in vitro in a DC-like phenotype [24]. Extrapolation from animal to human remains challenging due to poor species overlap notably concerning toll-like receptors (TLR) expression [25]. The aim of this work was to set up a method to assess the adjuvant or “danger like” effect of NP on human DCs.

We selected amorphous silica nanoparticles (aSNP) which have been poorly studied despite a large exposure of the population through drugs, food [26] or cosmetic products. In vitro studies showed that airway exposure to aSNP could enhance allergen sensitization, suggesting an adjuvant potential for these NP [27,28]. So far, most of the in vitro studies dedicated to aSNP remained limited to the phenotype analysis of DCs [6,23,29,30]. We showed that aSNP (12.5 µg·mL^−1^ and 25 µg·mL^−1^) increased the expression of CD83, CD86 and CXCR4 markers on human DCs in a concentration-dependent manner, suggesting that these aSNP significantly affected MoDCs maturation (Feray et al, in preparation). It is essential to consider the global adjuvant effect on the two first steps of the immune response, namely DCs activation leading to T-cell proliferation. Thus, we propose to complete these studies by developing an allogeneic co-culture model of human MoDCs and CD4+ T-cells.

This work describes the process to be followed in order to develop an in vitro model properly assessing the potential adjuvant effect of NP. We showed that the DC:T-cell ratios and the duration of the co-culture are critical parameters to correctly measure the influence of danger signals induced by NP in an allogeneic co-culture. To better visualize the effect of NP while keeping the basal proliferation inherent to the model as low as possible, we suggest testing at least three different ratios, preferably after five days of co-culture. We also recommend a careful selection of test concentrations based on realistic exposures and limiting cytotoxicity to maintain MoDCs function.

## 2. Materials and Methods

### 2.1. Reagents and Materials

According to the manufacturer, the specific surface of the fumed silica NP (S5505, Sigma-Aldrich, Saint-Louis, MO, USA), determined by BET (Brunauer-Emmett-Teller), is 196 m^2^.g^−^^1^ (batch SLBR6988V). The NP suspension was prepared from fumed silica dry powder dispersed in filtered ultrapure water at a final concentration of 50 mg·mL^−^^1^. The suspension was then left for 8 h in a water bath at 80 °C to remove contaminations. Before each experiment, an intermediate dilution at a concentration of 500 μg·mL^−^^1^ was prepared in Roswell Park Memorial Institute medium (RPMI) 1640 medium (supplemented with Glutamax, 1 mM sodium pyruvate, 0.1 ng·mL^−^^1^ streptomycin and 100 U·mL^−^^1^ penicillin). The suspension was sonicated for 15 min prior to MoDCs treatment.

### 2.2. Characterization of aSNP

The hydrodynamic diameter of particles was determined at 25°C by Dynamic Light Scattering using a Zetasizer Nano ZS 90 (Malvern Instruments, Malvern, UK) operating at a fixed scattering angle at 90° and equipped with a Helium-Neon laser source with a wavelength of 633 nm. Measurements were performed in disposable 4 mL cuvettes with a 1 cm optical pathway and four optical faces (Sarstedt, Nümbrecht, Germany) containing an appropriate volume (1 mL) of the sample, prepared as described above, after dilution to 5 mg·mL^−^^1^ in the desired medium. The hydrodynamic diameter values were calculated using the Stokes-Einstein equation assuming a spherical shape of the particles. The particle size profile was obtained from the intensity-weighted distribution and the hydrodynamic diameter value corresponds to the median diameter derived from the cumulative distribution curve. The ζ-potential measurements were carried out with the same instrument at 25 °C by laser doppler velocimetry at a detection angle of 17° in DTS 1060 disposable cells (Malvern Instruments, Malvern, UK). Due to the ionic strength of the samples leading to high conductivity, measurements were performed using the monomodal mode and the Smoluchowski approximation was used to convert the electrophoretic mobility to ζ-potential. Values are given as mean values of ten measurements.

For transmission electron microscopy experiments, 2 μL of 1 mg·mL^−^^1^ aqueous suspension of NP was deposited onto a formvar/carbon coated copper grid and observed using a JEOL 1400 transmission electron microscopy (TEM) instrument (JEOL Ltd, Tokyo, Japan) operating at 120 kV. Analysis of recorded images was performed using the ImageJ software (1.52 version, National Institute for Health, Bethesda, MD, USA).

### 2.3. Endotoxin Detection

Possible endotoxin contamination (endotoxins > 0.05 EU·mL^−^^1^) was analyzed with the limulus amebocyte lysate (LAL) assay (GenScript, Piscataway, NJ, USA).

### 2.4. Generation of Primary Cultures of Human Dendritic Cells

Human DCs were derived from monocytes isolated from human peripheral blood supplied by the French Blood Bank (EFS, Rungis, France). Healthy donors gave their written consent for the use of blood donation for research purposes. Peripheral blood mononuclear cells (PBMCs) were sorted from buffy coats by density centrifugation on a Ficoll gradient. Monocytes were then isolated through positive magnetic selection using MidiMacs separation columns and anti-CD14+ antibodies coated on magnetic beads (Miltenyi Biotec, Bergisch Gladbach, Germany). Finally, CD14 cells were differentiated in immature MoDCs for 4 days in RPMI 1640 supplemented with GlutaMAX (Gibco, Invitrogen, Saint Aubin, France), 10 % heat-inactivated fetal calf serum (FCS, Gibco, Invitrogen, Saint Aubin, France), 550 U·mL^−^^1^ granulocyte-macrophage colony-stimulating factor (rh-GM-CSF, Miltenyi Biotec, Bergisch Gladbach, Germany), 550 U·mL^−^^1^ interleukin-4 (rh-IL4, Miltenyi Biotec, Bergisch Gladbach, Germany), 1 mM sodium pyruvate (Gibco, Invitrogen, Saint Aubin, France), 100 µg·mL^−^^1^ streptomycin and 100 U·mL^−^^1^ penicillin (Gibco, Invitrogen, Saint Aubin, France).

### 2.5. Phenotypic Analysis

After 16 h of exposure to either 0, 12.5 or 25 µg·mL^−^^1^ aSNP, MoDCs were collected, washed with phosphate-buffered salin (PBS, Gibco, Invitrogen, Saint Aubin, France) and incubated for 20 min at 4 °C with APC-conjugated HLA-DR monoclonal antibody (mAbs) or with appropriate isotype control antibody (IgG1-APC) (BD Biosciences, Le Pont de Claix, France). After incubation, MoDCs were washed with PBS and then analyzed on a FACS Attune NxT^®^ acoustic focusing cytometer (ThermoFisher Scientific, Waltham, MA, USA). Results were expressed as the relative fluorescence intensities (RFI) using the corrected mean fluorescence intensity (cMFI) as follows:

(1) $$cMFI=MFI-MFI_{control\;isotype}$$

(2) $$RFI=\frac{cMFI_{treated\;cells}\;}{cMFI_{untreated\;cells}\;}$$

### 2.6. Co-Culture of MoDCs and CD4+ T-Cells

**Figure 1:**
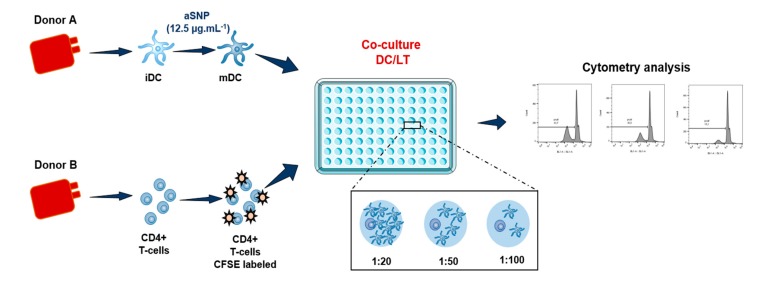
Allogeneic model of DCs and CD4 + T-cells co-culture with the different ratios tested. Cells are incubated in presence or absence of 12.5 μg·mL^−1^ of aSNP for 16 h. Treated MoDCs are co-cultured with allogeneic CD4+ T-cells labeled with CFSE at a ratio of 1:20 (1 MoDC for 20 CD4+ T-cells), 1:50 and 1:100). Proliferation is quantified after 5 or 6 days of co-culture as the percentage of CFSE^low^ CD4+ T-cells.

CD4+ T cells were isolated from PBMC by positive selection using midi-MACS separation columns and anti-CD4 antibodies coated on magnetic beads (Miltenyi Biotec, Bergisch Gladbach, Germany) (Figure 1). These T-cells were confirmed to have a purity greater than 95%, based on CD4 expression evaluated by flow cytometry (561841, RPA-T4, BD Biosciences, Le Pont de Claix, France). CD4+ T lymphocytes were labeled with 0,5 mM carboxyfluorescein succinimidyl ester (CFSE) (Invitrogen, Saint Aubin, France), following the manufacturer’s instructions. MoDCs were stimulated by 0 or 12.5 µg·mL^−^^1^ of aSNP for 16 h and then washed and co-cultured with allogeneic CD4+ T-cells at different DC:T-cell ratios for 5 or 6 days, in RPMI 1640 Glutamax supplemented with 10% FCS in round-bottom 96 well plates (Cellstar, Greiner bio-one, Courtaboeuf, France) (Figure 1). The number of T-cells (10^5^ T-cells/well) remained unchanged while the number of DCs evolved for each ratio tested: 1:5 (1 DCs for 5 T-cells i.e., 20 000 MoDCs/well), 1:10 (10 000 MoDCs/well), 1:20 (5000 MoDCs/well), 1:50 (2000 MoDC/well) and 1:100 (1000 MoDC/well). Analysis of T-cell proliferation was assessed by flow cytometry, on a FACS Attune NxT® acoustic focusing cytometer (ThermoFisher Scientific, Waltham, MA, USA).

## 3. Results and Discussion

### 3.1. Characterization of Amorphous Silica Nanoparticles

**Figure 2:**
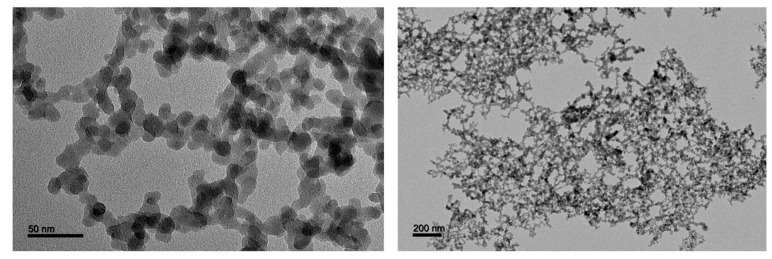
Typical TEM images of unstained fumed silica nanoparticles suspended in water. Aqueous suspension of nanoparticles was deposited onto a formvar/carbon coated copper grid and observed using a JEOL 1400 TEM instrument operating at 120 kV. Analysis of recorded images was performed using ImageJ 1.52 software.

We used commercial amorphous silica NP supplied by Sigma–Aldrich (S5505). This fumed silica nanopowder is presented by the manufacturer as having an average primary particle size of 14 nm and a surface area of 200 ± 25 m^2^·g^−1^. A precise characterization of the size, spherical shape and homogeneity of the fumed silica in water was carried out by transmission electron microscopy (TEM). The particles form branched, three-dimensional chain-like aggregates of variable lengths (Figure 2). The main characteristics of aSNP used in this study are summarized in Table 1.

Endotoxin levels were systematically measured for each particle preparation with the limulus amebocyte lysate (LAL) assay to ensure that the effects measured were not attributable to this contaminant [31]. All the batches tested (*n* = 8) showed values below the threshold of positivity (0.05 EU.mL^−1^).

### 3.2. Assessing the Effects of Amorphous Silica Nanoparticules on the Expression of Major Histocompatibility Complex Class II and co-stimulatory Molecules in Human Dendritic Cells

We conducted this study using human DCs, which represent the most efficient antigen presenting cells in capturing, processing and presenting antigens for lymphocyte activation. To avoid interspecies variability, we chose human MoDCs, which can be differentiated in vitro in a DC-like phenotype in the presence of granulocyte-macrophage colony-stimulating factor (GM-CSF) and IL-4 [24]. The GM-CSF in vitro cultured DCs is the most common and recognized DCs type used in studies of human DCs biology. These cells show similarities in physiology, morphology, and function to conventional myeloid DCs.

Immature DCs (iDCs) display a phenotype characterized by low surface expression of MHC-II molecules and co-stimulatory molecules such as CD80, CD86 and CD40 [32]. When detecting molecular patterns identified as danger signals, DCs undergo a program of maturation resulting in morphological and phenotypical changes. If in vitro immature DCs exhibit a round shape and a smooth membrane, mature DCs gain a rough surface with multiple dendrites and pseudopodia [33], as shown in Figure S1 in presence of aSNP. Moreover, scattering of light can be used to measure volume (by forward scatter) and morphological complexity (by side scatter) of cells by flow cytometry. These parameters are conventionally abbreviated as FSC and SSC respectively. Representative FSC and SSC density plots are presented on Figure S1b and illustrate a slight increase in SSC in presence of aSNP or with LPS, consistent with MoDCs activation. MHC-II and co-stimulatory molecules are upregulated at the cell surface. New interleukin and chemokine receptors are also expressed allowing T-cell stimulation and improving their ability to migrate to secondary lymphoid tissues [11].

Our goal was first to define the appropriate readouts and settings to evaluate MoDCs maturation. In a preliminary work, we selected the incubation time and concentrations to be tested based on cytotoxicity assays. Considering that NP could have the potential to alter immune cell responses at concentrations far below those inducing cytotoxicity, and that functional assays need to be conducted on viable cells, we defined a threshold of 30% cytotoxicity not to be exceeded. Given the cytotoxicity of aSNP and the short moDCs lifespan in vitro, incubation times longer than 24 h wouldn’t be achievable. Indeed, following their activation, DCs are known to progress toward apoptotic cell death. Typical incubation times to assess DCs phenotype range from overnight (approximately 16 h) to 24 h [6,29]. We chose a 16-h incubation time which allowed to evidence a significant effect of NP on DCs phenotype while complying with the 30% cytotoxicity threshold limit, in order to maintain their ability to activate an effective immune response.

**Figure 3:**
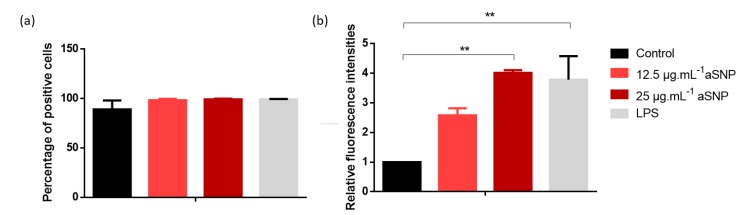
Nanoparticles induced HLA-DR expression. Cells were incubated in the presence or absence of LPS as a positive control, or 12.5 and 25 μg·mL^−1^ of aSNP for 16 h. Cells were then collected, washed and the surface expression of HLA-DR was assessed by FACS analysis. Untreated DCs were used as negative control. Results are expressed as percentage of positive cells (**a**) or relative fluorescence intensities (**b**) and represented the mean ± SEM of three independent experiments. Tukey’s honest significance test was employed in conjunction with One-Way ANOVA **: *p* < 0.01.

According to the literature, the expression of co-stimulatory molecules such as CD40, CD80 and CD86 is frequently used as a marker of successful MoDCs maturation [7,20,21,22,23]. These markers have also emerged as the most relevant in our experiences. CD83 is recognized as a surface marker that distinguishes immature and mature human DCs populations and is considered as one of the best markers for identifying mature dendritic cells [34]. HLA-DR can also be used as an indicator of the iDC maturation process. Interestingly, the expression of the results as a percentage of positive cells (Figure 3a) may not allow to evidence the increase in HLA-DR expression, as also observed by Vallhov et al [21]. A high expression of MHC-II molecules is inherent to the MoDCs model [11]. Hence, it appears necessary to present the results as RFIs instead of the percentage of positive cells (Figure 3b). Indeed, RFIs allowed us to show that aSNP induced a significant increase in MHC-II molecules expression at 25 µg·mL^−1^ (Figure 3b).

We established, using the panel presented above, that aSNP (12.5 µg·mL^−1^ and 25 µg·mL^−1^) dose-dependently increased the expression of CD83, CD86 and CXCR4 markers on human MoDCs (Feray et al, in preparation), suggesting that cells could be in a relatively mature state after the incubation with NP.

### 3.3. Assessing the Effect of Dendritic Cells Treated with Amorphous Silica Nanoparticules on Allogeneic T-Cell Proliferation

#### 3.3.1. Influence of DC:T-Cell Ratios on T-Cell Proliferation

Due to their phenotypic modifications, mature MoDCs can become immunogenic and promote the proliferation of CD4+ T-cells. To evaluate the impact of MoDCs maturation on the lymphocyte response, we developed a human mixed lymphocyte reaction (MLR) assay consisting in co-culturing CD4+ T-cells with allogeneic MoDCs. The MLR provides a simple and efficient in vitro model in the area of cellular immunology for the study of T-cell activation and proliferation. Specifically, we tested several DC:T-cell ratios in order to optimize the sensitivity of the method. Indeed, although co-stimulatory molecules and cytokines are known to be a prerequisite for productive DC-T-cell interactions, the ratio of stimulating DCs and responding T-cells is also known to influence T-cell responses in vitro [35].

**Figure 4:**
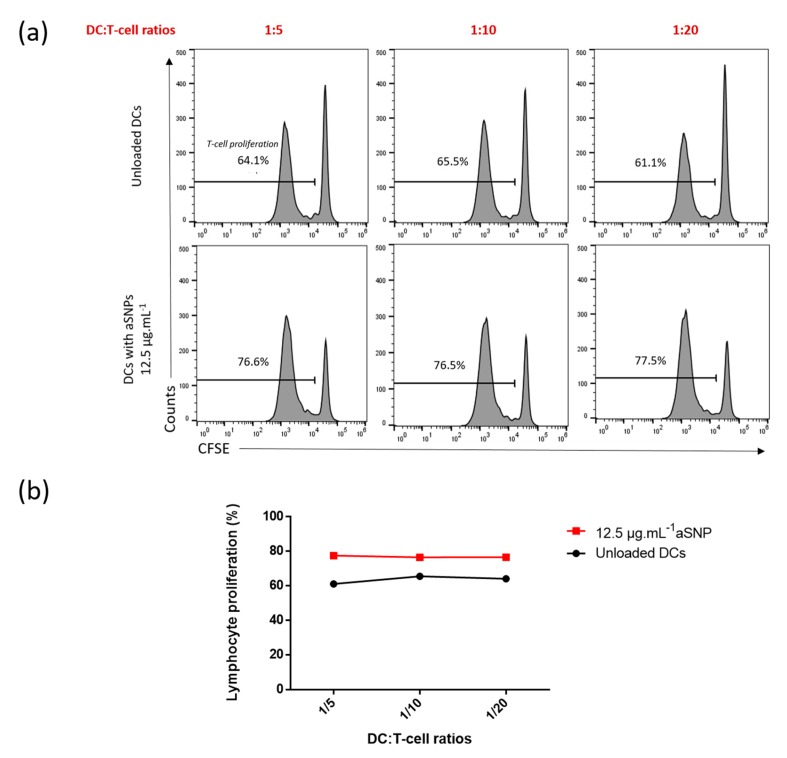
Lymphocyte proliferation reached a plateau at high ratios after 6 days of co-culture. Cells were incubated in the presence or absence of 12.5 μg·mL^−1^ of aSNP for 16 h. Treated MoDCs were co-cultured with allogeneic CD4+ T-cells labeled with CFSE at the ratios of 1:5 (1 MoDC per 5 CD4+ T-cells), 1:10 and 1:20). Proliferation was quantified after 6 days of co-culture as the percentage of CFSE^low^ CD4+ T-cells. Untreated DCs were used as negative control. Results are shown as percentage of lymphocyte proliferation using FACS histogram (**a**) or curve (**b**) for a representative experiment.

To avoid excessive cytotoxicity, MoDCs were stimulated or not with the lowest concentration of aSNP (12.5 µg·mL^−1^) for 16 h and then washed and co-cultured with allogeneic CD4+ T-cells labeled with carboxyfluorescein succinimidyl ester (CFSE) at various DC:T-cell ratios. Proliferation was assessed after six days of co-culture as the percentage of CFSE^low^ CD4+ T-cells (Figure 4). We first evaluated the influence of the following DC:T-cell ratios: 1:5 (corresponding to 1 MoDC per 5 T-cells), 1:10 (1 MoDC per 10 T-cells) and 1:20 (1 MoDC per 20 T-cells) (Figure 4).

Allogeneic MoDCs induced basal T-cell proliferation ranging from 61.1% to 64.1% at the highest ratio of 1:5 (Figure 4a). In presence of aSNP, the T-lymphocyte proliferation slightly increased, reaching a maximal value around 77%, regardless of the ratio tested (Figure 4). We could clearly visualize (Figure 4b) that at the high ratios of 1:5 and 1:10, T-cell proliferation reached a plateau. These results suggest that, considering the nature of the DCs stimulation, a high stimulator/responder ratio leads to a saturation in T-cell proliferation masking the NP effect, as also observed with carbon black NP [19]. In the following experiments, we decided to decrease DC:T-cell ratios, to reduce T-cell proliferation below this saturation threshold and increase the sensitivity of the method.

**Figure 5:**
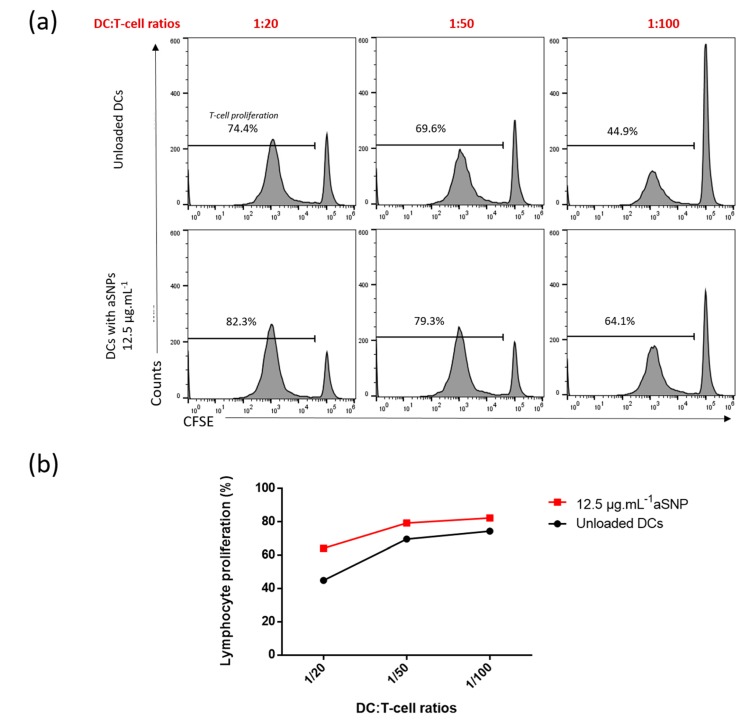
Low ratios allowed a better visualization of the increase in lymphocyte proliferation after 6 days of co-culture. Cells were incubated in the presence or absence of 12.5 μg·mL^−1^ of aSNP for 16 h. Treated MoDCs were co-cultured with allogeneic CD4+ T-cells labeled with CFSE at a ratio of 1:20 (1 moDC for 20 CD4+ T-cells), 1 :50 and 1 :100). Proliferation was quantified after 6 days of co-culture as the percentage of CFSE^low^ CD4+ T-cells. Untreated DCs were used as negative control. Results are shown as percentage of lymphocytes proliferation using FACS histogram (**a**) or curve (**b**) for a representative experiment.

To tackle this issue, we kept the 1:20 ratio that emerged as the most appropriate in the previous experience and added the 1:50 (1 MoDC per 50 T-cells) and 1:100 (1 MoDC per 100 T-cells) ratios (Figure 5). First, we observed that reducing the DC:T-cell ratios from 1:20 to 1:100 decreased the basal proliferation from 74.4% to 44.9% (Figure 5a). With the latter value being far below the saturation threshold, the effect of NP on T-cell proliferation was better assessed. Indeed, at the ratio of 1:20 the NP-induced proliferation increase was about 7.9% whereas it reached 19.2% for the 1:100 ratio (Figure 5a). With this latter ratio, the sensitivity noticeably increased (Figure 5b).

Altogether, these results underline the importance of systematically testing at least three ratios for each experiment. These observations also demonstrate the interest of low ratios. There are only few studies using the co-culture of T-cells and DCs to assess the functional DCs maturation in presence of NP, concerning carbon black [19] and CeO_2_/TiO_2_ [23] NP. Koike et al. [19] demonstrated that murine BM-DC treated with carbon black NP could enhance T-cell proliferation in a MLR-test. The authors used two ratios: 1.25% (2.5 × 10^3^ DC for 2 × 10^5^ T cells) and 2.5% (5 × 10^3^ DC for 2 × 10^5^ T cells) which reached a saturation threshold, especially for 56 nm carbon black NP [19]. A third ratio, for example 5%, would have probably increased the sensitivity of the method. Schanen et al. [23] used human DCs and naive T-cells with a single optimized ratio of 1:400. They observed that TiO_2_ NP had a modest immunostimulatory effect on T-cells, leading them to the co-administration of the mitogens PHA and PMA, to increase the proliferative response. This low response to the NP alone, which could be attributed to the use of naïve T-cell, would have warranted, as suggested above, to test more ratios including a higher relative amount of stimulator cells.

#### 3.3.2. Influence of the co-Culture Duration on T-Cell Proliferation and Assessment of Nanoparticles Effects

**Figure 6:**
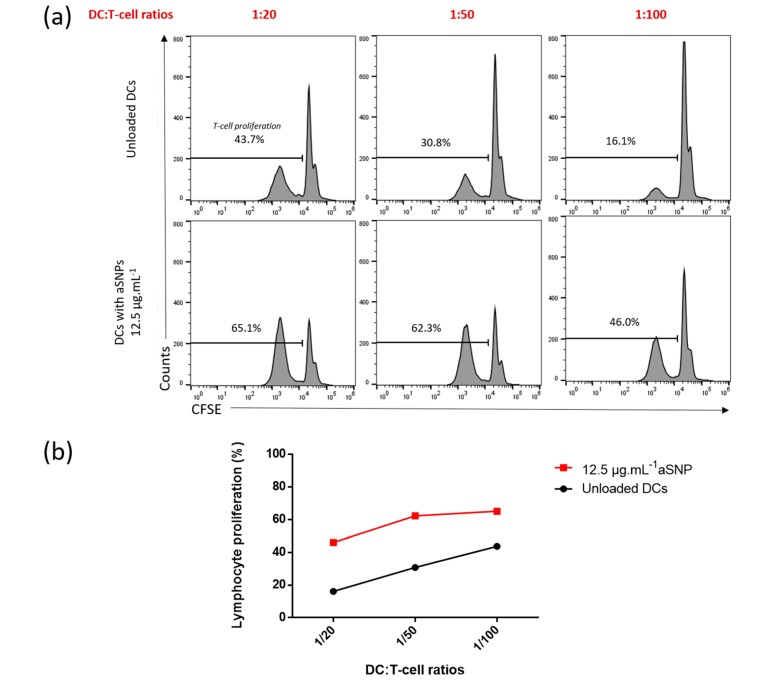
aSNP induced an increase in lymphocyte proliferation after 5 days of co-culture. Cells were incubated in the presence or absence of 12.5 μg·mL^−1^ of aSNP for 16 h. Treated MoDCs were co-cultured with allogeneic CD4+ T-cells labeled with CFSE at ratios of 1:20 (1 moDC for 20 CD4+ T-cells), 1:50 and 1:100). Proliferation was quantified after 5 days of co-culture as the percentage of CFSE^low^ CD4+ T-cells. Untreated DCs were used as negative control. Results are shown as percentage of lymphocyte proliferation using FACS histogram (**a**) or curve (**b**) for a representative experiment.

Although the interaction between DCs and T-cells under physiological conditions is unlikely to be as static and long lasting as in cell culture plates, the in-depth review of the literature led us to test 2 different co-culture durations which are commonly used to study T cell activation by the mixed-leukocyte-reaction [22,23,36]. As suggested above, reducing the basal T-cell proliferation improved the sensitivity of the model. To further decrease this proliferation, we decided to reduce the co-culture duration from six to five days (Figure 6). We retained the three ratios selected above which proved to be effective in detecting NP-induced T-cell proliferation (1:100, 1:50 and 1:20). Interestingly, in comparison to what was observed at day 6 (D6), allogeneic MoDCs induced a lower basal proliferation at day 5 (D5), ranging from 16.1 to 43.7% (Figure 6a). The ratio 1:100 allowed to substantially reduce the proliferation to 16.1% after five days of co-culture compared to 44.9% after six days. Consequently, T-cells showed a strong proliferative response when MoDCs were stimulated with aSNP (Figure 6a and Figure 5b). If the NP-induced proliferation increase was already observable after 6 days of co-culture (Figure 5), it seemed clearly more pronounced after five days (Figure 6b), reaching a value of about 30% for both ratios 1:50 and 1:100. The five days co-culture setting allowed to significantly increase the sensitivity for detecting NP effects using this co-culture model.

**Figure 7:**
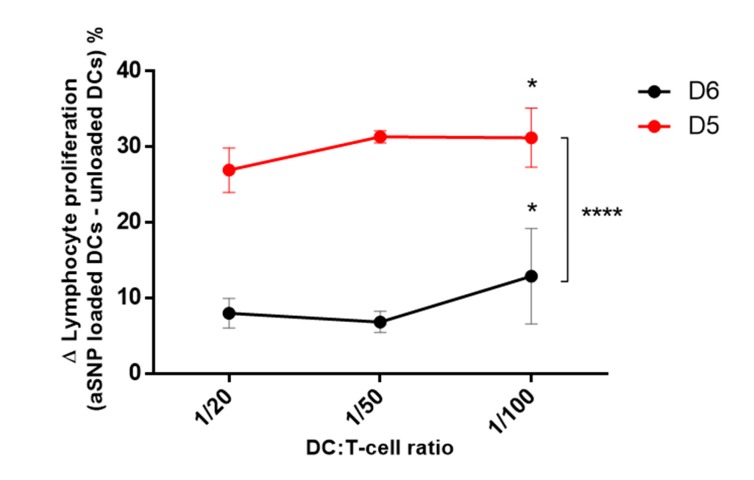
The 5-days incubation period is optimal to highlight lymphocyte proliferation augmentation induced by aSNP. Cells were incubated in the presence or absence of 12.5 μg.mL^−1^ of aSNP for 16 h. Treated MoDCs were co-cultured with allogeneic CD4+ T-cells labeled with CFSE at ratios of 1:20 (1 moDC for 20 CD4+ T-cells), 1 :50 and 1 :100). Proliferation was quantified after 5 or 6 days of co-culture as the percentage of CFSE^low^ CD4+ T-cells. Untreated DCs were used as negative control. Results are showed as the Δ (aSNP loaded DC-unloaded DCs), corresponding to the proliferation of NP-treated DCs-proliferation of untreated DCs, and represent the mean ± SEM of three independent experiments. Tukey’s honest significance test was employed in conjunction with Two-way ANOVA *: statistically significant compared to 1:20 ratio with *p* < 0.05, **** : *p* < 0.0001.

To better visualize the effect of NP in our experiments, we calculated the differences between proliferations obtained with unloaded DCs and with aSNP-loaded DCs, after five (D5) or six days (D6) of co-culture. The results obtained in three independent experiments are shown in Figure 7. We observed that the differences in proliferation were evidenced in both culture conditions, albeit at a higher level at D5 than at D6, confirming the results showed previously in a representative experiment (Figure 6). This way of representing the results is particularly illustrative of the improvement brought by the reduction in co-culture time. Moreover, these results emphasize once again the importance of considering a range of ratios. To conclude, we recommend selecting the five-days co-culture conditions, and to test at least three different ratios to assess the adjuvant effect of NP.

The present study demonstrates that the MLR-induced T-cell proliferation assay is suitable for the evaluation of the immunomodulatory potential of NP. Nevertheless, the main drawback when using ex vivo differentiated human primary DCs is the donor-to-donor variability [8]. The heterogeneity of haplotypes between DCs and T-cells donors may lead to elevated basal proliferation that can even mask the adjuvant effect of NP. Moreover, in addition to unavoidable variations in cell yield, we clearly observed a donor-dependent fluctuation in MoDCs activation, depending on the donor’s health, quality of life and even seasons. For all these reasons, it was essential to optimize the experimental conditions to minimize this variability, particularly by reducing the co-culture time and the DC:T-cell ratios. It appears also essential to carry out the experiments on at least three to five different donors.

## 4. Conclusions

Our human allogeneic model of co-culture allows to show the pro-adjuvant effect of aSNP at the two first steps of the immune response: DCs activation and T-cell proliferation. These findings highlight the importance of assessing NP effects on immune cell function beyond the standard endpoints such as oxidative stress or cytotoxicity. As a result, it seems essential to complete the phenotypic characterization of DCs maturation by a functional test exploring their ability to promote the proliferation of CD4+ T-cells. We propose a five-days co-culture including a wide range of ratios, from 1:5 to 1:100, in order to minimize basal proliferation values and to avoid the proliferation saturation that would prevent the effect of NP from being detected.

## Figures and Tables

**Table 1:** Characteristics of aSNP.

Reference	Specific Surface Area ^1^	Nominal Primary Particle Diameter ^1^	Primary Particle Diameter ^2^	DLS Distribution ^3^	*ζ*-Potential Value ^3^
S5505, Sigma-Aldrich (batch SLBR6988V)	196 m^2^.g^−1^	14 nm	14.4 ± 4.3 nm	201 ± 22 nm 9.0 ± 0.3 µm	−26.5 ± 2.1 mV

^1^ Data provided by the supplier, ^2^ Analyzed by TEM, ^3^ in RPMI 1640

## Supplementary Materials

The following are available online at https://www.mdpi.com/2079-4991/10/3/425/s1, Figure S1. Morphological and dimensional modifications of dendritic cells in presence of amorphous silica nanoparticles.
