# Supplementary material for: How to Address the Adjuvant Effects of Nanoparticles on the Immune System
Source: Nanomaterials (Basel). 2020 Feb 28;10(3):425. doi: 10.3390/nano10030425 (PMC7152845; doi:10.3390/nano10030425)
Supplement: Supplementary file 1 [file nanomaterials-10-00425-s001.pdf]

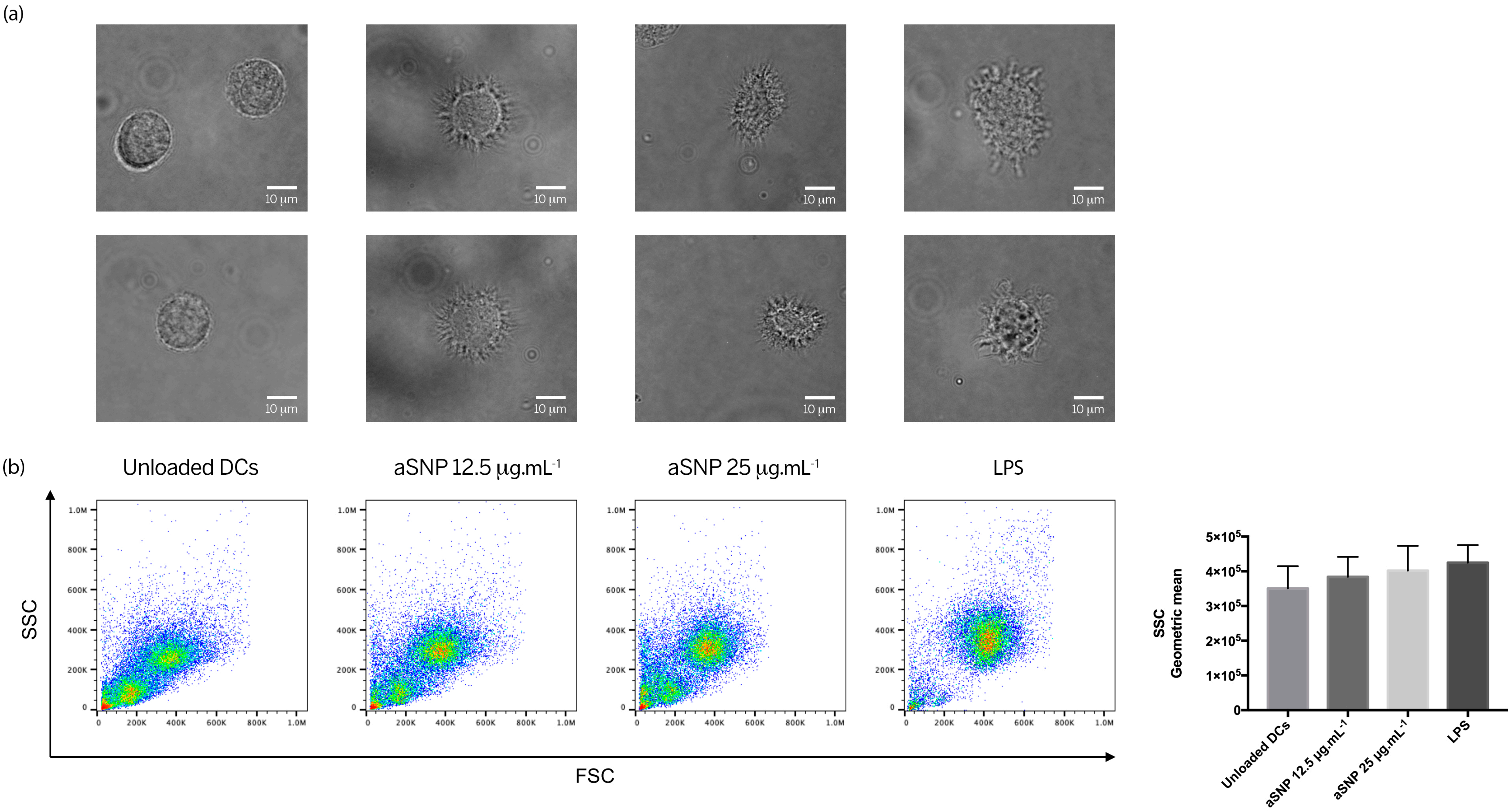

Figure S1. Morphological and dimensional modifications of dendritic cells in presence of amorphous silica nanoparticles.
(a) Cells were incubated in the presence or absence of LPS as a positive control, or 12.5 and 25 µg.mL-1 of aSNP for 16 hours. Cells were then collected, washed with PBS and analyzed with the Nikon Eclipse 280i microscope using the x100 objective.
(b) Cells were treated as in (a) and analysed by FACS. Representative FSC and SSC density plots. The results of four independent experiments are presented on the right panel.
